# Chikungunya seroprevalence in population-based studies: a systematic review and meta-analysis

**DOI:** 10.1186/s13690-023-01081-8

**Published:** 2023-05-01

**Authors:** Lacita Menezes Skalinski, Aline Elena Sacramento Santos, Enny Paixão, Martha Itaparica, Florisneide Barreto, Maria da Conceição Nascimento Costa, Maria Glória Teixeira

**Affiliations:** 1 Universidade Estadual de Santa Cruz, Rodovia Jorge Amado, km 16, s/n, Salobrinho, Ilhéus, CEP 45662-900 BA Brasil; 2 Instituto de Saúde Coletiva/ Universidade Federal da Bahia, Rua Basílio da Gama, s/n, Campus Canela, Salvador, CEP 40110-040 BA Brazil; 3 London School of Hygiene and Tropical Medicine, Keppel St, London, WC1E 7HT UK

**Keywords:** Chikungunya Virus, Seroepidemiologic Studies, Health Surveys

## Abstract

**Background:**

Seroprevalence studies about chikungunya infection are usually conducted after epidemics to estimate the magnitude of the attack. This study aimed to estimate the seroprevalence of CHIKV by WHO region, considering the periods of introduction of the virus in these regions and its potential to lead to epidemics.

**Methods:**

We systematically reviewed Medline/Pubmed, Embase, Lilacs, Scopus and Web of Science for original articles published up to 2020. Cohort, case-control and cross-sectional studies were eligible for inclusion, based on the results of laboratory diagnosis of previous or previous and recent infection. Those conducted with symptomatic individuals were excluded.

**Results:**

596 articles were identified, 197 full-text were reviewed and 64 were included, resulting in 71 seroprevalences. Most were cross-sectional studies (92%), between 2001 and 2020 (92%), with population of all ages (55%), conducted in Kenya (10.9%), Brazil (9.4%) and French Polynesia (7.8%). The pooled estimates were 24% (95%CI 19–29; I^2^ = 99.7%; p < 0.00), being 21% (95%CI 13–30; I^2^ = 99.5%; p < 0.00) for adults, 7% (95%CI 0–23; I^2^ = 99.7%; p < 0.00) for children and 30% (95%CI 23–38; I^2^ = 99.7%; p < 0.00) for all ages. The higher seroprevalences were found in African, the Americas and South-East Asian Regions.

**Conclusions:**

The great heterogeneity of seroprevalences points to the persistence of viral circulation. Even where the seroprevalence is high, the population replacement and the absence of vaccines mean that the risk of virus spread and epidemics remains.

**Registration:**

PROSPERO CRD42020166227.

**Supplementary Information:**

The online version contains supplementary material available at 10.1186/s13690-023-01081-8.

## Background

Chikungunya is an arbovirus caused by an alphavirus, Chikungunya virus (CHIKV), which is transmitted by the bite of *Aedes* genus mosquitos. It was isolated for the first time in 1952, when it was responsible for an outbreak in Tanzania [1]. In the 2000s, CHIKV emerged as an important infectious disease when epidemics of great magnitude broke out in Kenya, where the attack rate was 75% of the population [2]. From 2004 to 2006, the rapid spread of CHIKV resulted in more than 500,000 cases reported in the surrounding regions of the Indian Ocean and in La Reunion Island, where 35% of the population was infected [3]. Since then, epidemics have occurred in India, Africa and Europe [4], and in 2013, CHIKV was introduced in the Americas, with an explosive epidemic in Saint Martin [5] and by 2021 it had led to more than 6.5 million cases in the Americas WHO Region [6]. Since then, studies have shown the emergence of chronic and disabling forms, giving rise to clinical and epidemiological concern among scientists and health authorities [7].

Seroprevalence studies about CHIKV infection are usually conducted after epidemics to estimate the magnitude of the attack rate, identifying the proportion of asymptomatic cases [8, 9]. Furthermore, these studies elucidate the diagnosis given that there is a confusion with other urban arboviruses that cocirculate in the same space, especially in countries where the laboratory support is inadequate [10].

The potential to provoke epidemics, chronic and disabling forms, together with the absence of vaccines and the difficulties of control measures highlight the importance of scientific knowledge about the real burden of chikungunya, in addition to identifying naive populations and the herd immunity. This systematic review and meta-analysis therefore aims to estimate the seroprevalence of CHIKV by WHO region, considering the periods of introduction of the virus in these regions and its potential to lead to epidemics.

## Methods

This Systematic Review and Meta-analysis research was conducted following the guidelines of the Preferred Reporting Items for Systematic Reviews and Meta-analysis (PRISMA) [11] and registered in the database of Prospective Register of Systematic Reviews (PROSPERO) under number CRD42020166227.

### Search strategy and eligibility criteria

The data were extracted from studies included in Medline/Pubmed, Embase, Lilacs, Scopus and Web of Science databases, without language restriction and published until December 31st, 2020. The descriptors used on the search were “Chikungunya Virus”, “Chikungunya Fever”, “Seroepidemiologic Studies”, “Health Surveys” and “Surveys and Questionnaires”. These descriptors were combined with boolean operators “OR” and “AND” to identify the studies that might be included on this systematic and meta-analysis review. Duplicates were removed and then the eligibility criteria were applied.

The eligible studies were those which presented the seroprevalence of chikungunya in populations in observational studies, including case-control, cross-sectional and cohort studies, based on the results of laboratory diagnosis of previous or previous and recent infection by antibodies detection (Elisa IgG, Elisa IgG + IgM and/or molecular diagnosis, immunofluorescence – IF, hemagglutination inhibition – HI, neutralization - NT). Studies that only presented results indicating recent or acute infections were excluded, as well as those conducted with symptomatic individuals in health services turned to investigation of febrile illness. Case report studies and reviews were used as sources of references only and were not considered in this systematic review. The specificity and sensitivity of the tests were not considered for analysis.

### Study selection and data collection

Four reviewers (L.M.S., A.E.S.S., F.B. and M.I.) worked in pairs to screen the titles and abstracts that fulfilled the criteria, which were then read completely. The pairs extracted data filling in a standardized form, which contained the following information: author, journal and year of publication, country and city, period, study design, populational group, sample, age, laboratorial method and number of positive results. Whenever disagreements occurred, they were resolved by consensus.

### Risk of bias assessment

The quality of articles was evaluated by two reviewers (L.M.S. and A.E.S.S.), using the critical appraisal tools for use in systematic reviews [12]. Nine criteria guided the analysis, including aspects of selection, representativeness and description of population, sample size, coverage and availability of diagnosis methods, statistical analysis and management of low response rate. Each criterion was answered with yes, no, unclear or not applicable. Each “yes” was considered a point in the evaluation and the higher the number of yes, the lower the risk of bias. The ones with 7 to 9 points were considered as low risk of bias, between 4 and 6 as moderate and 1 to 3 as high risk.

### Statistical analysis

The outcome considered in this study was the seroprevalence of CHIKV among populations and its 95% Confidence Intervals (95% CI). The seroprevalence was calculated with the number of positive cases, evidenced by lab tests, divided by the number of people who were tested.

The seroprevalence was estimated by the population groups recruited (less than 15 years old - children, more than 15 years old – adults and studies with all ages – general population) and by World Health Organization Regions (African, Americas, Eastern Mediterranean, European, South-East Asian and Western Pacific) which the countries in the studies belong to.

In order to perform meta-analysis of proportions, metapropp was applied in Stata Software. Metapropp calculates the 95% Confidence Intervals (95% CI) using the score statistic and the exact binomial method, incorporating Freeman-Tukey double arcsine transformation of proportions and models the variability using the binomial distribution [13].

The weights were calculated to represent the size of the contribution of each individual study to the average of seroprevalence. Heterogeneity (I^2^) was used to express the variability among studies in the systematic review and to explain whether this variability can be randomly attributed. The I^2^ values less than 50% indicate absence or moderate variability, while I^2^ higher than 50% may represent substantial heterogeneity. If it is higher than 75%, this indicates considerable heterogeneity. Chi square test was applied to evaluate the significance of I^2^, considering the level of p < 0.10 [14].

## Results

**Fig. 1:**
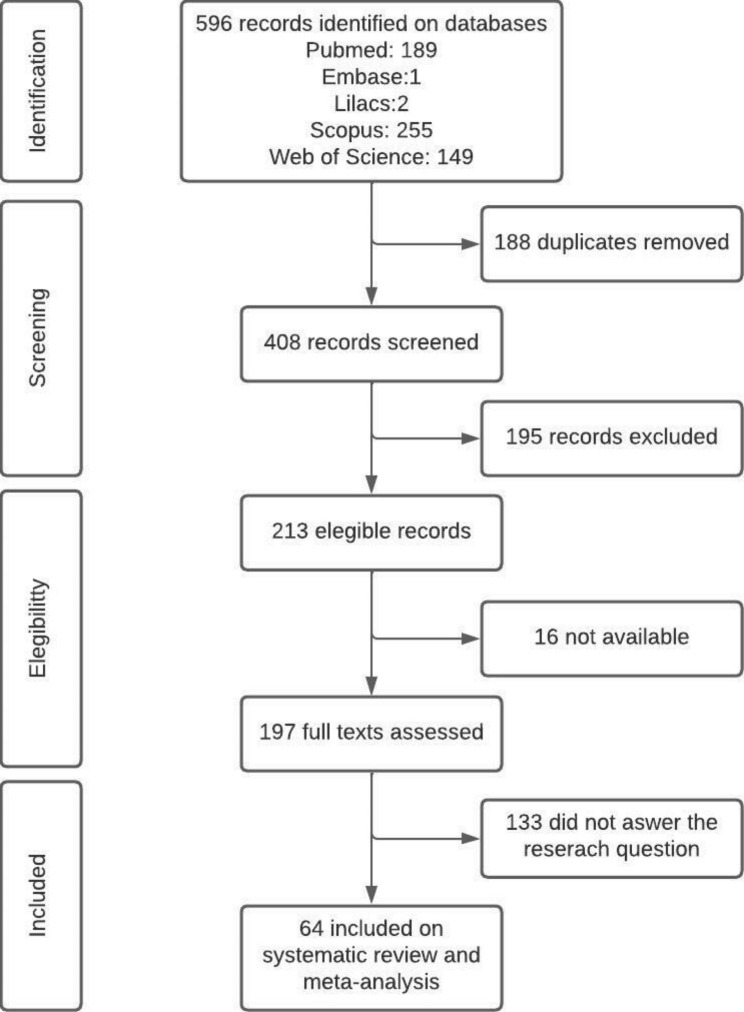
Flowchart of study selection for systematic review and meta-analysis

After searching the databases, 596 articles were identified. Of these, 188 were removed because they were duplicates, leaving 408 articles that had their titles and abstracts read. A total of 213 articles were considered eligible, but 16 of them were not available to access (Supplementary material 1). Thus, 197 articles were read in full and 133 did not answer the study question. Then 64 articles were included in the systematic review (Fig. 1, Supplementary material 2). Six publications presented more than one population study, so we considered them separately in the analysis, finding 71 results of seroprevalence.

Most of the studies (92.2%) were published between 2001 and 2020 and were conducted in Kenya (10.9%), Brazil (9.4%) and French Polynesia (7.8%). Cross sectional was the study design used in 92.3% of publications and 70.4% had samples of 1,000 participants or fewer. Regarding the population of study, 54.9% were of all ages, 33.8% of studies recruited only adults (> 15 years old) and 11.3% were composed of only children (< 15 years old). Elisa tests were performed in 77% of publications (Table 1, Supplementary material 2).

**Table 1:** Characteristics of seroprevalence studies of chikungunya virus included in the systematic review and meta-analysis, 1960–2020

Characteristic	n	%
**Year of publication**		
1960–1980	4	6.3
1981–2000	1	1.5
2001–2020	59	92.2
**Country of study**		
Kenya	7	10.9
Brazil	6	9.4
French Polynesia	5	7.8
India	4	6.3
Comoros	4	6.3
Cameroon	2	3.1
Fiji	2	3.1
Nicaragua	2	3.1
Thailand	2	3.1
United States	2	3.1
Others	28	43.8
**Study design**		
Cross sectional	60	92.3
Cohort	3	4.7
Cohort and cross sectional	1	1.5
Case Control	1	1.5
**Sample size**		
75-1000	50	70.4
1001–2000	13	18.3
≥ 2001	8	11.3
**Population of study**		
General population (all ages)	39	54.9
Adults	24	33.8
Children	8	11.3
**Risk of bias (points)**		
Low (7–9)	30	46.9
Moderate (4–6)	23	35.9
High (1–3)	11	17.2

The risk of bias was classified as low in 46.9%, moderate in 35.9% and high in 17.2% of the studies (Table 1, Supplementary material 3). Analyzing the nine criteria separately, the worst in evaluation were the adequate sample size, which was present only in 36.9% of the studies, followed by the sufficient coverage of the identified sample (52.3%) and sample frame appropriate to address the target population (55.4%).

The overall seroprevalence identified in the 71 studies was 24% (95%CI 19–29), with high heterogeneity (I^2^ = 99.7%; p < 0.00) in meta-analysis.

**Fig. 2:**
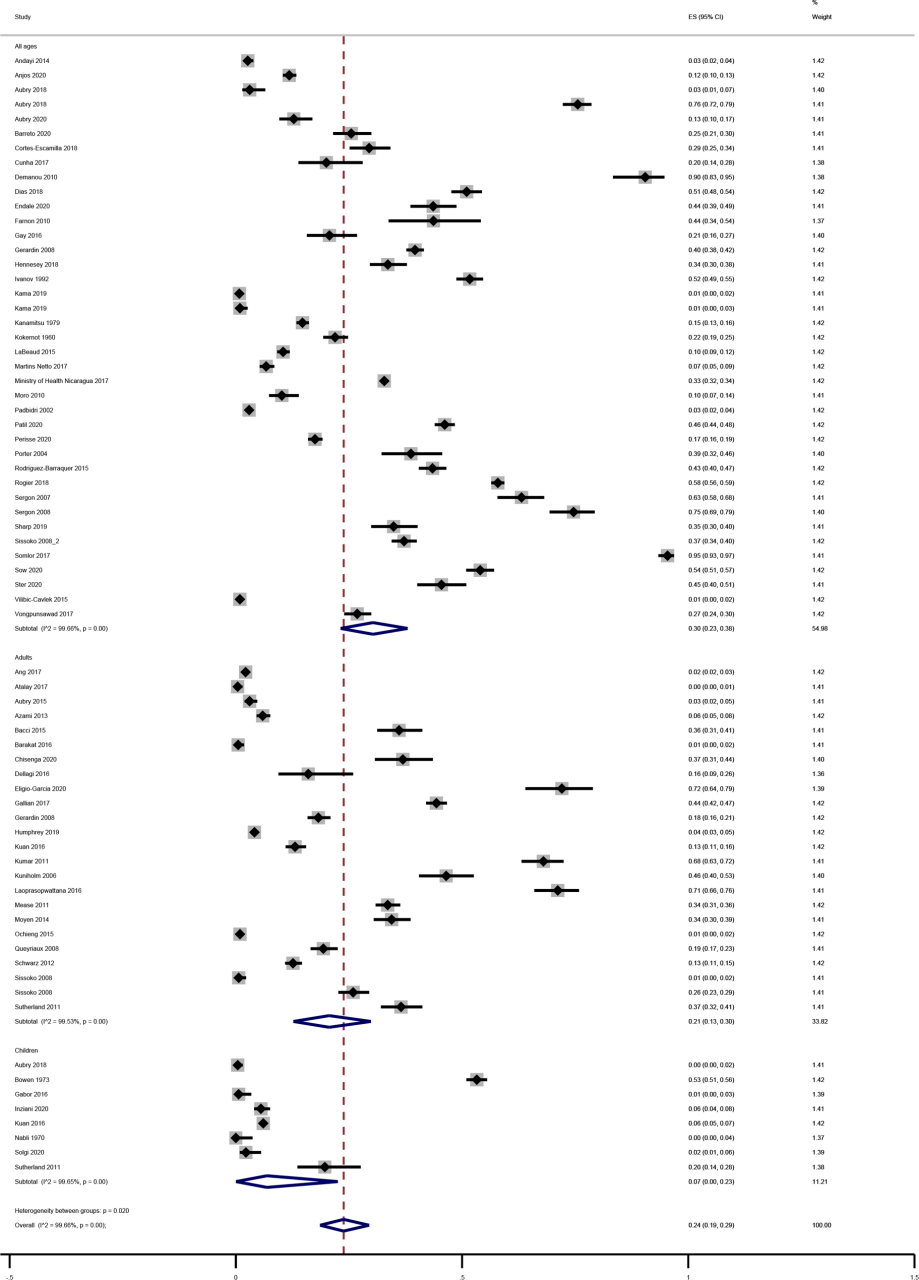
Forest plot showing the results of the meta-analysis by author and population group

The seroprevalence of CHIKV in adults was calculated based on 24 studies [3, 15–36]. The pooled seroprevalence in this set of studies was 21% (95%CI 13–30), with high heterogeneity (I^2^ = 99.5%; p < 0.00) observed in meta-analysis. The lowest seroprevalence observed was 0.4% (95%CI 0.1–1.5) in Turkey [15] and the highest was 71.2% (95%CI 66.0-75.9) in Thailand [27] (Fig. 2).

Eight studies were conducted only with children [18, 29, 37–42] and the pooled seroprevalence was 7% (95%CI 0–23), with high heterogeneity (I^2^ = 99.7%; p < 0.00). No cases were identified in Tunisia [41], the lowest seroprevalence was 0.2 (95%CI 0.1–1.2) in French Polynesia [37] and the highest was 53.3% (95%CI 50.9–55.6) in Kenya [38] (Fig. 2).

Thirty-nine studies included populations of all ages [2, 28, 37, 43–76], with pooled seroprevalence of 30% (95%CI 23–38), with high heterogeneity (I^2^ = 99.7%; p < 0.00). The lowest seroprevalence observed was 0.8% (95%CI 0.4–1.7) in Fiji [49] and the highest was 95.4% (95%CI 93.4–96.9) in Laos [64] (Fig. 2).

Analyzing the seroprevalence by WHO Region, the highest one was African, where the seroprevalence found was 31% (95%CI 21–41), followed by Americas, with 29% (95%CI 19–39) and South-East Asian, with 24% (95%CI 19–29)(Table 2, Supplementary material 4).

**Table 2:** Number of studies, pooled seroprevalence, Confidence Interval of 95% (95%CI) and heterogeneity (I^2^) in meta-analysis by WHO Region

Region	N of studies	Seroprevalence	95% CI	I^2^ (%)*
African	4	31	21–41	99.43
Americas	15	29	19–39	99.68
Eastern Mediterranean	6	5	1–10	96.21
European	4	5	0–17	99.88
South-East Asian	7	24	19–29	99.73
Western Pacific	15	18	7–32	99.78

* p-value was < 0.00 in all heterogeneity tests

Considering only the studies of low risk of bias, the pooled seroprevalence was 27% (95%CI 19–36), with high heterogeneity (I^2^ = 99.7%; p < 0.00), and the studies of low and moderate risk showed a pooled seroprevalence of 25% (95%CI 20–31), with high heterogeneity (I^2^ = 99.6%; p < 0.00) (data not shown).

## Discussion

This study revealed that the overall seroprevalence of CHIKV among the 71 population serosurveys was 24%. The highest seroprevalence found was 95.4% in a study conducted in Laos [64], where 542 of 568 participants from the general population had at least one positive immunoglobulin isotype (IgM and/or IgG) and the lowest was 0.2% in a study conducted with 476 children in French Polynesia [37], with only IgG tests. This great variability was also found in studies conducted only with adults, which indicated 21% of pooled seroprevalence that varied from 0.4 to 71.2%. In those surveys that included only children the pooled seroprevalence was 7%, varying from 0.2 to 53.3%. Similarly, in the population of all ages the variation was from 0.8 to 95.4%, with pooled seroprevalence of 30%. African was the WHO Region with the highest seroprevalence (31%), followed by Americas (29%) and South-East Asian (24%).

In a systematic review conducted by other authors which included studies published from 2000 to 2019, the overall seroprevalence of CHIKV was 25% (95% CI: 22–29) [77], similar to what we found in this review, even considering a shorter and more recent time period. This must be due to the circulation of CHIKV that was more restricted to Africa and Southeast Asia in the period prior to 2000. It included 44 studies and the South-East Asian Region had the highest seroprevalence among all WHO regions (42%, 95% CI: 17–67), followed by African Region (33%, 95% CI: 24–41) [77] In our study, African showed the highest seroprevalence, probably because we included studies before 2000. As expected, a scope review including publications of studies carried out from 1989 to 2017 also evidenced great variability (0.4–76.0%) in 54 studies [78].

In our review there were studies in 38 countries located in all the WHO Regions, however, they do not represent these Regions as a whole, since the virus circulation has already been identified in 114 countries [79]. There was great seroprevalence variability among WHO regions, 31% in Africa, followed by the Americas with 29%, and the lowest was 5% in the Eastern Mediterranean and European regions. Although there were no studies of all the 114 countries, it is plausible to hypothesize that this variability is real, because the results depict the higher or lower intensity and time of circulation of CHIKV in each Region to a certain extent.

All surveys used similar laboratory tests that identify the presence of antibodies against CHIKV. However, the specificity and sensitivity of the tests were not considered in the analysis, which may have biased the study results. Furthermore, the methods for selecting the population of the surveys were diverse and the sample size calculation was not always described in the methods section, as evidenced by the qualitative analysis of the articles included in this meta-analysis. These differences in methods inevitably lead to statistical heterogeneity of meta-analyses, which generally include a small number of studies which constitutes a limitation, given that the power of the heterogeneity test in these circumstances is low [80]. Another important factor is that in prevalence studies with large sample sizes and narrow confidence intervals, the heterogeneity result can be misleading [81, 82].

We understand that the great heterogeneity between the seroprevalence values found here is not surprising and cannot be attributed only to the aforementioned reasons, insofar as the level of herd immunity produced by arboviruses is modulated by several factors that show different characteristics in distinct areas. In fact, in addition to the infectivity power of the agent, the time of introduction and circulation in a given population also play a role in the greater or lesser receptivity of the environment to the vector. This receptivity, with regard to CHIKV transmitters, is determined not only by climatic conditions but also by the environmental sanitation infrastructure, the living conditions of the populations and the availability and implementation of control measures. All these factors will condition the population density of transmitting mosquitoes in each area. Furthermore, it is also necessary to consider the density of the human population in urban centers, as it is a very important factor in this process [83, 84].

The value of 95.4%, the highest seroprevalence observed in our review, verified in a survey conducted in Laos, a year after the first laboratorial evidence of CHIKV circulation in this country [85] can be partly explained by its location in a region of tropical climate and rainy seasons that favor the reproduction of the vector. However, this impressive find could be the result of the intense and previous circulation of CHIKV in this country, as highlighted by Somlor et al. in 2017 [64]. On the other hand, the 90% seroprevalence found in Cameroon is not surprising, as CHIKV is known to have been circulating there since at least 2001 [86], as evidenced in a survey conducted from 2000 to 2003, where the seroprevalence was already 46% [22]. These high levels of seroprevalence indicate that CHIKV had been circulating previously in both countries. If in fact this arbovirus had been introduced in Laos in 2012, the seroprevalence would possibly have been much lower.

The study with French Polynesian children was carried out between May and June 2014 in Tahiti, its most populous island. At the beginning of the chikungunya epidemic in this archipelago the study found a seroprevalence of 0.2% [37]. However, right after the outbreak of this epidemic, in another survey carried out from September to November 2015, including only adults, the seroprevalence reached more than 75.6% [37], revealing the force of infection of this arbovirus. However, this is not the only factor to consider. For example, although there is a similarity in terms of climate and living conditions between Fiji and French Polynesia, the epidemic did not explode at the same rate in the two countries, which can be seen from the seroprevalence that was only 13% [72]. Perhaps this difference is due to the cross-protection generated by infections produced by the Ross River Virus, an agent that circulates in Fiji and belongs to the same group as CHIKV [72], producing febrile clinical conditions and polyarthritis.

It is a fact that CHIKV has been circulating in the African and South-East Asian Regions since 1950s [1, 87]. Since then, CHIKV has produced periodic outbreaks for approximately 50 years, until the occurrence of the 2005 epidemic in the Indian Ocean Islands [28, 62].This prolonged circulation may partly explain this high pooled seroprevalence, in addition to the human replacement of naïve population and the high *Ae. aegypti* and *Ae. albopictus* densities in these Regions. In the Americas, this arbovirus only emerged in 2013 [88] and the high level of seroprevalence can be a result of the speed of dissemination and high epidemic levels that many populous cities in several countries have seen since then. This is probably due to the widespread distribution and high population density of both vectors, especially in large urban centers, which had already favored the occurrence of successive epidemics of the four serotypes of the DENV since the 1980s. The emergence of CHIKV and ZIKV have worsened this epidemiological situation, and despite all the efforts that many countries alongside PAHO are implementing to reduce the population of these mosquitoes, the results have mostly been inadequate. This scenario points to the urgent need to develop new technologies to control these urban arboviruses, not only for vector control, but especially for vaccines for the populations.

## Conclusions

In countries with an abundance of vectors and naive populations, the persisting viral circulation remains an epidemiological concern and must be a target for surveillance and control measures. Even where the seroprevalence is high, the human population replacement, the absence of vaccines to prevent the infection by CHIKV and the low effectiveness of currently available vector control measures, the risk of virus spread remains and the possible occurrence of epidemics.

## Electronic supplementary material

Below is the link to the electronic supplementary material.

Supplementary Material 1

Supplementary Material 2

Supplementary Material 3

Supplementary Material 4

## Data Availability

All data generated or analysed during this study are included in this published article and its supplementary information files.

## References

[1] Ross RW (1956). The Newala epidemic: III. The virus: isolation, pathogenic properties and relationship to the epidemic. J Hyg (Lond).

[2] Sergon K, Njuguna C, Kalani R, Ofula V, Onyango C, Konongoi LS (2008). Seroprevalence of Chikungunya Virus (CHIKV) infection on Lamu Island, Kenya, October 2004. Am J Trop Med Hyg.

[3] Sissoko D, Moendandze A, Malvy D, Giry C, Ezzedine K, Louis J (2008). Seroprevalence and risk factors of Chikungunya Virus infection in Mayotte, Indian Ocean, 2005–2006: a Population-Based survey. PLoS ONE.

[4] Johansson MA, Powers AM, Pesik N, Cohen NJ, Erin Staples J. Nowcasting the spread of Chikungunya Virus in the Americas.PLoS One. 2014;9(8). 10.1371/journal.pone.0104915 PMC4128737 25111394

[5] Leparc-Goffart I, Nougairede A, Cassadou S, Prat C, Lamballerie X. Chikungunya in the Americas. Lancet [Internet]. 2014;383. Available from: https://www.thelancet.com/journals/lancet/article/PIIS01406736(14)60185-9/fulltext 10.1016/S0140-6736(14)60185-9 24506907

[6] Pan American Health Organization. World Health Organization. Cases of Chikungunya Virus Disease [Internet]. [cited 2022 Mar 13]. Available from: https://www3.paho.org/data/index.php/en/mnu-topics/chikv-en/550-chikv-weekly-en.html

[7] Paixão ES, Rodrigues LC, Costa M, da CN, Itaparica M, Barreto F, Gérardin P (2018). Chikungunya chronic disease: a systematic review and meta-analysis. Trans R Soc Trop Med Hyg.

[8] Staples JE, Breiman RF, Powers AM. Chikungunya Fever: An Epidemiological Review of a Re-Emerging Infectious Disease. Clin Infect Dis [Internet]. 2009 Sep 15;49(6):942–8. Available from: https://academic.oup.com/cid/article-lookup/doi/10.1086/605496 10.1086/605496 19663604

[9] Teo T-H, Her Z, Tan JJL, Lum F-M, Lee WWL, Chan Y-H (2015). Caribbean and La Réunion Chikungunya Virus isolates Differ in their capacity to induce proinflammatory Th1 and NK cell responses and Acute Joint Pathology. J Virol.

[10] Oliveira JF, Rodrigues MS, Skalinski LM, Santos AES, Costa LC, Cardim LL et al. Interdependence between confirmed and discarded cases of dengue, chikungunya and Zika viruses in Brazil: A multivariate time-series analysis.PLoS One. 2020;15(2). 10.1371/journal.pone.0228347 PMC6996800 32012191

[11] Page MJ, McKenzie JE, Bossuyt PM, Boutron I, Hoffmann TC, Mulrow CD et al. The PRISMA 2020 statement: An updated guideline for reporting systematic reviews.BMJ. 2021;372. 10.1136/bmj.n71 PMC8005924 33782057

[12] Munn Z, MClinSc SM, Lisy K, Riitano D, Tufanaru C (2015). Methodological guidance for systematic reviews of observational epidemiological studies reporting prevalence and cumulative incidence data. Int J Evid Based Healthc.

[13] Nyaga VN, Arbyn M, Aerts M, Metaprop (2014). A Stata command to perform meta-analysis of binomial data. Arch Public Heal.

[14] Cochrane Training. Cochrane Handbook for Systematic Reviews of Interventions [Internet]. [cited 2022 Mar 18]. Available from: https://training.cochrane.org/handbook/current

[15] Atalay T, Kaygusuz S, Azkur AK (2017). A study of the chikungunya virus in humans in Turkey. Turkish J Med Sci.

[16] Moyen N, Thiberville SD, Pastorino B, Nougairede A, Thirion L, Mombouli JV (2014). First reported chikungunya fever outbreak in the republic of Congo, 2011. PLoS ONE.

[17] Bacci A, Marchi S, Fievet N, Massougbodji A, Perrin RX, Chippaux JP (2015). High seroprevalence of chikungunya virus antibodies among pregnant women living in an urban area in Benin, West Africa. Am J Trop Med Hyg.

[18] Sutherland LJ, Cash AA, Huang YJS, Sang RC, Malhotra I, Moormann AM (2011). Serologic evidence of arboviral infections among humans in Kenya. Am J Trop Med Hyg.

[19] Chisenga CC, Bosomprah S, Musukuma K, Mubanga C, Chilyabanyama ON, Velu RM et al. Sero-prevalence of arthropod-borne viral infections among Lukanga swamp residents in Zambia. Samy AM, editor. PLoS One [Internet]. 2020 Jul 1;15(7):e0235322. Available from: 10.1371/journal.pone.0235322 10.1371/journal.pone.0235322 PMC7329080 32609784

[20] Humphrey JM, Al-Absi ES, Hamdan MM, Okasha SS, Al-Trmanini DM, El-Dous HG (2019). Dengue and chikungunya seroprevalence among qatari nationals and immigrants residing in Qatar. PLoS ONE.

[21] Gallian P, Leparc-Goffart I, Richard P, Maire F, Flusin O, Djoudi R (2017). Epidemiology of Chikungunya Virus Outbreaks in Guadeloupe and Martinique, 2014: an observational study in Volunteer Blood Donors. PLoS Negl Trop Dis.

[22] Kuniholm MH, Wolfe ND, Huang CYH, Mpoudi-Ngole E, Tamoufe U, Burke DS (2006). Seroprevalence and distribution of Flaviviridae, Togaviridae, and Bunyaviridae arboviral infections in rural cameroonian adults. Am J Trop Med Hyg.

[23] Azami NAM, Salleh SA, Shah SA, Neoh H, Othman Z, Zakaria SZS et al. Emergence of chikungunya seropositivity in healthy Malaysian adults residing in outbreak-free locations: Chikungunya seroprevalence results from the Malaysian Cohort. BMC Infect Dis [Internet]. 2013 Dec 5;13(67). Available from: https://bmcinfectdis.biomedcentral.com/articles/10.1186/1471-2334-13-67 10.1186/1471-2334-13-67 PMC3651385 23379541

[24] Eligio-García L, Crisóstomo-Vázquez MDP, Caballero-García M, de L, Soria-Guerrero M, Méndez–galván JF, López-Cancino SA et al. Co-infection of dengue, zika and chikungunya in a group of pregnant women from tuxtla gutiérrez, chiapas: Preliminary data. 2019. PLoS Negl Trop Dis. 2020;14(12):1–11. 10.1371/journal.pntd.0008880 PMC7785221 33347432

[25] Kumar NP, Suresh A, Vanamail P, Sabesan S, Krishnamoorthy KG, Mathew J (2011). Chikungunya virus outbreak in Kerala, India, 2007: a seroprevalence study. Mem Inst Oswaldo Cruz.

[26] Barakat AM, Smura T, Kuivanen S, Huhtamo E, Kurkela S, Putkuri N (2016). The presence and seroprevalence of arthropod-borne viruses in Nasiriyah Governorate, Southern Iraq: a cross-sectional study. Am J Trop Med Hyg.

[27] Laoprasopwattana K, Suntharasaj T, Petmanee P, Suddeaugrai O, Geater A (2016). Chikungunya and dengue virus infections during pregnancy: Seroprevalence, seroincidence and maternal-fetal transmission, southern Thailand, 2009–2010. Epidemiol Infect.

[28] Gérardin P, Guernier V, Perrau J, Fianu A, Le Roux K, Grivard P (2008). Estimating Chikungunya prevalence in La Réunion Island outbreak by serosurveys: two methods for two critical times of the epidemic. BMC Infect Dis.

[29] Kuan G, Ramirez S, Gresh L, Ojeda S, Melendez M, Sanchez N et al. Seroprevalence of Anti-Chikungunya Virus Antibodies in Children and Adults in Managua, Nicaragua, After the First Chikungunya Epidemic, 2014–2015. Bingham A, editor. PLoS Negl Trop Dis [Internet]. 2016 Jun 20;10(6):e0004773. Available from: 10.1371/journal.pntd.0004773 10.1371/journal.pntd.0004773 PMC4913910 27322692

[30] Ochieng C, Ahenda P, Vittor AY, Nyoka R, Gikunju S, Wachira C et al. Seroprevalence of Infections with Dengue, Rift Valley Fever and Chikungunya Viruses in Kenya, 2007. Kehn-Hall K, editor. PLoS One [Internet]. 2015 Jul 15;10(7):e0132645. Available from: 10.1371/journal.pone.0132645 10.1371/journal.pone.0132645 PMC4503415 26177451

[31] Dellagi K, Salez N, Maquart M, Larrieu S, Yssouf A, Silaï R (2016). Serological evidence of contrasted exposure to Arboviral Infections between Islands of the Union of Comoros (Indian Ocean). PLoS Negl Trop Dis.

[32] Schwarz NG, Girmann M, Randriamampionona N, Bialonski A, Maus D, Krefis AC (2012). Seroprevalence of antibodies against chikungunya, dengue, and Rift Valley fever viruses after febrile illness outbreak, Madagascar. Emerg Infect Dis.

[33] Ang LW, Kam YW, Lin C, Krishnan PU, Tay J, Ng C (2017). Seroprevalence of antibodies against chikungunya virus in Singapore resident adult population. PLoS Negl Trop Dis.

[34] Queyriaux B, Armengaud A, Jeannin C, Couturier E, Peloux-Petiot F (2008). Chikungunya in Europe. Lancet.

[35] Aubry M, Finke J, Teissier A, Roche C, Broult J, Paulous S (2015). Seroprevalence of arboviruses among blood donors in french polynesia, 2011–2013. Int J Infect Dis.

[36] Mease LE, Coldren RL, Musila LA, Prosser T, Ogolla F, Ofula VO et al. Seroprevalence and distribution of arboviral infections among rural Kenyan adults: A cross-sectional study.Virol J. 2011;8(July). 10.1186/1743-422X-8-371 PMC3161961 21794131

[37] Aubry M, Teissier A, Huart M, Merceron S, Vanhomwegen J, Mapotoeke M (2018). Seroprevalence of dengue and chikungunya virus antibodies, french Polynesia, 2014–2015. Emerg Infect Dis.

[38] Bowen ETW, Simpson DIH, Platt GS, Way H, Bright WF, Day J (1973). Large scale irrigation and arboviruses epidemiology, Kano Plain, Kenya. II. Preliminary serological survey. Trans R Soc Trop Med Hyg.

[39] Gabor JJ, Schwarz NG, Esen M, Kremsner PG, Grobusch MP. Dengue and chikungunya seroprevalence in Gabonese infants prior to major outbreaks in 2007 and 2010: A sero-epidemiological study. Travel Med Infect Dis [Internet]. 2016;14(1):26–31. Available from: 10.1016/j.tmaid.2016.01.005 10.1016/j.tmaid.2016.01.005 26869532

[40] Inziani M, Adungo F, Awando J, Kihoro R, Inoue S, Morita K et al. Seroprevalence of yellow fever, dengue, West Nile and chikungunya viruses in children in Teso South Sub-County, Western Kenya. Int J Infect Dis [Internet]. 2020;91:104–10. Available from: 10.1016/j.ijid.2019.11.004 10.1016/j.ijid.2019.11.004 31712089

[41] Nabli B, Chippaux-Hyppolite C, Chippaux A, Tamalet J (1970). Enquête sérologique en tunisie sur les arbovirus. Bull World Health Organ.

[42] Solgi A, Karimi A, Armin S. Seropositivity of Chikungunya and West Nile Viruses in Iranian Children in 2018. Arch Pediatr Infect Dis [Internet]. 2020 Apr 28;8(2). Available from: http://pedinfect.com/en/articles/94416.html

[43] Dias JP, Costa MCN, Campos GS, Paixão ES, Natividade MS, Barreto FR et al. Seroprevalence of Chikungunya Virus after Its Emergence in Brazil. Emerg Infect Dis [Internet]. 2018 Apr;24(4):617–24. Available from: https://wwwnc.cdc.gov/eid/article/24/4/pdfs/17-1370.pdf 10.3201/eid2404.171370 PMC5875253 29553317

[44] Endale A, Michlmayr D, Abegaz WE, Asebe G, Larrick JW, Medhin G et al. Community-based sero-prevalence of chikungunya and yellow fever in the South Omo Valley of Southern Ethiopia. PLoS Negl Trop Dis [Internet]. 2020;14(9):e0008549. Available from: 10.1371/journal.pntd.0008549 10.1371/journal.pntd.0008549 PMC7470273 32881913

[45] Farnon EC, Gould LH, Griffith KS, Osman MS, El Kholy A, Brair ME (2010). Household-Based sero-epidemiologic survey after a yellow fever epidemic, Sudan, 2005. Am J Trop Med Hyg.

[46] Gay N, Rousset D, Huc P, Matheus S, Ledrans M, Rosine J (2016). Seroprevalence of asian lineage chikungunya virus infection on saint martin island, 7 months after the 2013 emergence. Am J Trop Med Hyg.

[47] Hennessey MJ, Ellis EM, Delorey MJ, Panella AJ, Kosoy OI, Kirking HL (2018). Seroprevalence and symptomatic attack rate of chikungunya virus infection, United States virgin islands, 2014–2015. Am J Trop Med Hyg.

[48] Ivanov AP, Ivanova OE, Lomonosov NN, Pozdnyakov SV, Konstantinov OK, Bah MA. Serological investigations of Chikungunya virus in the Republic of Guinea. Vol. 72, Annales de la Société belge de médecine tropicale. 1992. p.73–4. 1567272

[49] Kama M, Aubry M, Naivalu T, Vanhomwegen J, Mariteragi-Helle T, Teissier A et al. Sustained Low-Level Transmission of Zika and Chikungunya Viruses after Emergence in the Fiji Islands. Emerg Infect Dis [Internet]. 2019 Aug;25(8):1535–8. Available from: http://wwwnc.cdc.gov/eid/article/25/8/18-0524_article.htm 10.3201/eid2508.180524 PMC6649350 31310218

[50] Kanamitsu M, Taniguchi K, Urasawa S, Ogata T, Wada Y, Wada Y (1979). Geographic distribution of arbovirus antibodies in indigenous human populations in the Indo-Australian archipelago. Am J Trop Med Hyg.

[51] Kokernot RH, Smithburn KC, Gândara AF, McIntosh BM, Heymann CS (1960). Provas de Neutralização com soros de indivíduos residentes em Moçambique contra determinados vírus isolados em África transmitidos por artópodes. An Inst Med Trop.

[52] Martins Netto E, Moreira-Soto A, Pedroso C, Höser C, Funk S, Kucharski AJ (2017). High Zika Virus Seroprevalence in Salvador, northeastern Brazil limits the potential of forther outbreaks. Am Soc Microbiol.

[53] Moro ML, Gagliotti C, Silvi G, Angelini R, Sambri V, Rezza G et al. Chikungunya Virus in North-Eastern Italy: A Seroprevalence Survey. 2010;82(3):508–11. 10.4269/ajtmh.2010.09-0322 PMC2829919 20207883

[54] Padbidri VS, Wairagkar NS, Joshi GD, Umarani UB, Risbud AR, Gaikwad DL (2002). A serological survey of arboviral diseases among the human population of the Andaman and Nicobar Islands, India. Southeast Asian J Trop Med Public Health.

[55] Patil HP, Rane PS, Gosavi M, Mishra AC, Arankalle VA (2020). Standardization of ELISA for anti-chikungunya-IgG antibodies and age-stratified prevalence of anti-chikungunya-IgG antibodies in Pune, India. Eur J Clin Microbiol Infect Dis.

[56] Périssé ARS, Souza-Santos R, Duarte R, Santos F, de Andrade CR, Rodrigues NCP et al. Zika, dengue and chikungunya population prevalence in Rio de Janeiro city, Brazil, and the importance of seroprevalence studies to estimate the real number of infected individuals. Roques P, editor. PLoS One [Internet]. 2020 Dec 17;15(12):e0243239. Available from: 10.1371/journal.pone.0243239 10.1371/journal.pone.0243239 PMC7746276 33332373

[57] Porter KR, Tan R, Istary Y, Suharyono W, Sutaryo, Widjaja S (2004). A serological study of Chikungunya virus transmission in Yogyakarta, Indonesia: evidence for the first outbreak since 1982. Southeast Asian J Trop Med Public Health.

[58] Rodríguez-Barraquer I, Solomon SS, Kuganantham P, Srikrishnan AK, Vasudevan CK, Qbal SH (2015). The hidden burden of dengue and chikungunya in chennai, India. PLoS Negl Trop Dis.

[59] Rogier EW, Moss DM, Mace KE, Chang M, Jean SE, Bullard SM (2018). Use of bead-based serologic assay to evaluate chikungunya virus epidemic, Haiti. Emerg Infect Dis.

[60] Sergon K, Yahaya AA, Brown J, Bedja SA, Mlindasse M, Agata N (2007). Seroprevalence of Chikungunya virus infection on Grande Comore Island, Union of the Comoros, 2005. Am J Trop Med Hyg.

[61] Sharp TM, Lorenzi O, Torres-Velásquez B, Acevedo V, Pérez-Padilla J, Rivera A (2019). Autocidal gravid ovitraps protect humans from chikungunya virus infection by reducing Aedes aegypti mosquito populations. PLoS Negl Trop Dis.

[62] Sissoko D, Malvy D, Giry C, Delmas G, Paquet C, Gabrie P (2008). Outbreak of Chikungunya fever in Mayotte, Comoros archipelago, 2005–2006. Trans R Soc Trop Med Hyg.

[63] Andayi F, Charrel RN, Kieffer A, Richet H, Pastorino B, Leparc-Goffart I et al. A Sero-epidemiological Study of Arboviral Fevers in Djibouti, Horn of Africa.PLoS Negl Trop Dis. 2014;8(12). 10.1371/journal.pntd.0003299 PMC4263616 25502692

[64] Somlor S, Vongpayloth K, Diancourt L, Buchy P, Duong V, Phonekeo D et al. Chikungunya virus emergence in the Lao PDR, 2012–2013. Vol. 12,PLoS ONE. 2017. 10.1371/journal.pone.0189879 PMC5746231 29284012

[65] Sow A, Nikolay B, Faye O, Cauchemez S, Cano J, Diallo M et al. Changes in the Transmission Dynamic of Chikungunya Virus in Southeastern Senegal. Viruses [Internet]. 2020 Feb 10;12(2):196. Available from: https://www.mdpi.com/1999-4915/12/2/196 10.3390/v12020196 PMC7077306 32050663

[66] Chis Ster I, Rodriguez A, Romero NC, Lopez A, Chico M, Montgomery J (2020). Age-dependent seroprevalence of dengue and chikungunya: inference from a cross-sectional analysis in Esmeraldas Province in coastal Ecuador. BMJ Open.

[67] Vilibic-Cavlek T, Pem-Novosel I, Kaic B, Babić-Erceg A, Kucinar J, Klobucar A (2015). Seroprevalence and entomological study on chikungunya virus at the croatian littoral. Acta Microbiol Immunol Hung.

[68] Vongpunsawad S, Intharasongkroh D, Thongmee T, Poovorawan Y (2017). Seroprevalence of antibodies to dengue and chikungunya viruses in Thailand. PLoS ONE.

[69] Labeaud AD (2008). Why Arboviruses Can Be Neglected Tropical Diseases.

[70] Ministerio del Poder Ciudadano para la Salud de Nicaragua. Seroprevalencia y tasa de ataque clínica por chikungunya en Nicaragua, 2014–2015. Rev Panam Salud Publica [Internet]. 2017;41. Available from: http://iris.paho.org/xmlui/bitstream/handle/123456789/34103/v41a592017.pdf?sequence=1&isAllowed=y 10.26633/RPSP.2017.59 PMC6612730 28902272

[71] Anjos RO, Mugabe VA, Moreira PSS, Carvalho CX, Portilho MM, Khouri R (2020). Transmission of Chikungunya Virus in an Urban Slum, Brazil. Emerg Infect Dis.

[72] Aubry M, Kama M, Henderson AD, Teissier A, Vanhomwegen J, Mariteragi-Helle T (2020). Low chikungunya virus seroprevalence two years after emergence in Fiji. Int J Infect Dis.

[73] Barreto FK, de Alencar A, Araújo CH, de Oliveira FM, de MAB R, Cavalcante JW, Lemos DRQ et al. Seroprevalence, spatial dispersion and factors associated with flavivirus and chikungunya infection in a risk area: a population-based seroprevalence study in Brazil. BMC Infect Dis [Internet]. 2020;20(1):881. Available from: https://bmcinfectdis.biomedcentral.com/articles/10.1186/s12879-020-05611-5 10.1186/s12879-020-05611-5 PMC7685300 33234110

[74] Cortes-Escamilla A, López-Gatell H, Sánchez-Alemán M, Hegewisch-Taylor J, Hernández-Ávila M, Alpuche-Aranda CM (2018). The hidden burden of Chikungunya in central Mexico: results of a small-scale serosurvey. Salud Publica Mex.

[75] Cunha RV, Trinta KS, Montalbano CA, Sucupira MVF, Lima MM, Marques E et al. Seroprevalence of Chikungunya Virus in a Rural Community in Brazil.PLoS Negl Trop Dis. 2017;11(1). 10.1371/journal.pntd.0005319 PMC5287455 28107342

[76] Demanou M, Antonio-Nkondjio C, Ngapana E, Rousset D, Paupy C, Manuguerra JC et al. Chikungunya outbreak in a rural area of Western Cameroon in 2006: A retrospective serological and entomological survey.BMC Res Notes. 2010;3. 10.1186/1756-0500-3-128 PMC2883987 20444282

[77] Li Z, Wang J, Cheng X, Hu H, Guo C, Huang J et al. The worldwide seroprevalence of denv, chikv and zikv infection: A systematic review and meta-analysis. PLoS Negl Trop Dis [Internet]. 2021;15(4):1–17. Available from: 10.1371/journal.pntd.0009337 10.1371/journal.pntd.0009337 PMC8109817 33909610

[78] Fritzell C, Rousset D, Adde A, Kazanji M, Van Kerkhove MD, Flamand C. Current challenges and implications for dengue, chikungunya and Zika seroprevalence studies worldwide: A scoping review. PLoS Negl Trop Dis [Internet]. 2018;12(7):1–29. Available from: 10.1371/journal.pntd.0006533 10.1371/journal.pntd.0006533 PMC6062120 30011271

[79] Center for Disease Control and Prevention. Areas at Risk for Chikungunya. [Internet]. [cited 2022 Mar 5]. Available from: https://www.cdc.gov/chikungunya/geo/index.html

[80] Higgins JPT, Thompson SG, Deeks JJ, Altman DG (2003). Measuring inconsistency in meta-analyses. Br Med J.

[81] Rücker G, Schwarzer G, Carpenter JR, Schumacher M (2008). Undue reliance on I2 in assessing heterogeneity may mislead. BMC Med Res Methodol.

[82] Borenstein M, Higgins JPT, Hedges LV, Rothstein HR (2017). Basics of meta-analysis: I2 is not an absolute measure of heterogeneity. Res Synth Methods.

[83] Kuno G. Review of the factors modulating dengue transmission.Epidemiol Rev. 1995;17(2). 10.1093/oxfordjournals.epirev.a036196 8654514

[84] Teixeira MG, Barreto ML, Costa MCN, Ferreira LDA, Vasconcelos PF, Cairncross S (2002). Dynamics of dengue virus circulation: a silent epidemic in a complex urban area. Trop Med Int Heal.

[85] Chanthavy S, Phouthone S, Khonesavanh P, Darouny P, Sonesavanh P, Khamphaphongphane B et al. Emergence of chikungunya in Moonlapamok and Khong Districts, Champassak Province, the Lao People’s Democratic Republic, May to September 2012. West Pacific Surveill Response J [Internet]. 2013 Jan 13;4(1):46–50. Available from: http://ojs.wpro.who.int/ojs/index.php/wpsar/article/view/192/252 10.5365/WPSAR.2012.3.4.017 PMC3729106 23908956

[86] Ndip LM, Bouyer DH, Rosa APAT, Titanji VPK, Tesh RB, Walker DH. Acute Spotted Fever Rickettsiosis among Febrile Patients, Cameroon. Emerg Infect Dis [Internet]. 2004 Mar;10(3):432–7. Available from: http://wwwnc.cdc.gov/eid/article/10/3/02-0713_article.htm 10.3201/eid1003.020713 PMC3322773 15109409

[87] Wimalasiri-Yapa BMCR, Stassen L, Huang X, Hafner LM, Hu W, Devine GJ (2019). Chikungunya virus in Asia–Pacific: a systematic review. Emerg Microbes Infect.

[88] Cassadou S, Boucau S, Petit-Sinturel M, Huc P, Leparc-Goffart I, Ledrans M. Emergence of chikungunya fever on the French side of Saint Martin island, October to December 2013.Eurosurveillance. 2014;19(13). 10.2807/1560-7917.es2014.19.13.20752 24721536

