# Supplementary material for: Chikungunya seroprevalence in population-based studies: a systematic review and meta-analysis
Source: Arch Public Health. 2023 May 1;81:80. doi: 10.1186/s13690-023-01081-8 (PMC10150504; doi:10.1186/s13690-023-01081-8)
Supplement: Supplementary file 1 — Supplementary Material 1 [file 13690_2023_1081_MOESM1_ESM.docx]

**Additional file 1. Papers not found to integrate the systematic review**

| **Publication** | **Author** | **Title** | **Year** |
| --- | --- | --- | --- |
| J Egypt Public Health Assoc | Darwish MA, Imam IZ, Omar FM | A serological study of certain arbovirus antibodies in Egypt | 1974 |
| Bulletin of the World Health Organization | Poirier MJ, Moss DM, Feeser KR,  Streit TG, Chang G-JJ, Whitney M, et al. | Detection of immunoglobulin G responses in Haitian children exposed to chikungunya, dengue, and malaria using a multiplex bead assay | 2016 |
| Pahlavi Medical Journal | Saidi S | Survey of antibodies to arboviruses in human population of Iran | 1971 |
| J Egypt Public Health Assoc | Ibrahim SH, Darwish MA, Wahdan MH, el-Ghoroury AA | Serologic survey of Kuwait population for evidence of group A arbovirus infection | 1973 |
| Iran J Public Health | Saidi S | Viral antibodies in preschool children from the Caspian area, Iran | 1974 |
| J Trop Med Hyg | Omer AHS, McLaren ML, Johnson BK | A seroepidemiological survey in the Gezira, Sudan, with special reference to arboviruses | 1981 |
| Am J Trop Med Hyg | Halstead SB, Nimmannitya S, Margiotta MR | Dengue and chikungunya virus infection in man in Thailand, 1962-1964. II. Observations on disease in outpatients. | 1969 |
| Southeast Asian J Trap Med Public Health | Johnson DE, Scott RM, Nisalak A, Kenedy RS | Togavirus infection in rural Thailand | 1980 |
| Biken J | [Fukunaga](https://pubmed.ncbi.nlm.nih.gov/?term=Fukunaga+T&cauthor_id=4464834) T, [Rojanasuphot](https://pubmed.ncbi.nlm.nih.gov/?term=Rojanasuphot+S&cauthor_id=4464834) S,  [Pisuthipornkul](https://pubmed.ncbi.nlm.nih.gov/?term=Pisuthipornkul+S&cauthor_id=4464834) S, , [Wungkorbkiat](https://pubmed.ncbi.nlm.nih.gov/?term=Wungkorbkiat+S&cauthor_id=4464834) S, [Thammanichanon](https://pubmed.ncbi.nlm.nih.gov/?term=Thammanichanon+A&cauthor_id=4464834) A | Seroepidemiologic Study of Arbovirus Infections in the North-East and South of Thailand | 1974 |
| J Trop Med Hyg | [Morrill](https://pubmed.ncbi.nlm.nih.gov/?term=Morrill+JC&cauthor_id=2051522) JC, [Johnson](https://pubmed.ncbi.nlm.nih.gov/?term=Johnson+BK&cauthor_id=2051522) BK, [Hyams](https://pubmed.ncbi.nlm.nih.gov/?term=Hyams+C&cauthor_id=2051522) C,  [Okoth](https://pubmed.ncbi.nlm.nih.gov/?term=Okoth+F&cauthor_id=2051522) F,  [Tukei](https://pubmed.ncbi.nlm.nih.gov/?term=Tukei+PM&cauthor_id=2051522) PM, Mugambi M, Woody J | Serological Evidence of Arboviral Infections Among Humans of Coastal Kenya | 1991 |
| J Commun Dis | [Neogi](https://pubmed.ncbi.nlm.nih.gov/?term=Neogi+DK&cauthor_id=7636147) DK,  [Bhattacharya](https://pubmed.ncbi.nlm.nih.gov/?term=Bhattacharya+N&cauthor_id=7636147) N,  [Mukherjee](https://pubmed.ncbi.nlm.nih.gov/?term=Mukherjee+KK&cauthor_id=7636147) KK,  [Chakraborty](https://pubmed.ncbi.nlm.nih.gov/?term=Chakraborty+MS&cauthor_id=7636147) MS,  [Banerjee](https://pubmed.ncbi.nlm.nih.gov/?term=Banerjee+P&cauthor_id=7636147) P, [Mitra](https://pubmed.ncbi.nlm.nih.gov/?term=Mitra+K&cauthor_id=7636147) K, [Lahiri](https://pubmed.ncbi.nlm.nih.gov/?term=Lahiri+M&cauthor_id=7636147) M, [Chakravarti](https://pubmed.ncbi.nlm.nih.gov/?term=Chakravarti+SK&cauthor_id=7636147) SK | Serosurvey of Chikungunya Antibody in Calcutta Metropolis | 1995 |
| J Commun Dis | [Angami K](https://www.scopus.com/authid/detail.uri?authorId=6507987235&amp;eid=2-s2.0-0024678788), [Chakravarty SK,](https://www.scopus.com/authid/detail.uri?authorId=7102772760&amp;eid=2-s2.0-0024678788) [Das MS,](https://www.scopus.com/authid/detail.uri?authorId=7402050706&amp;eid=2-s2.0-0024678788) [Chakraborty MS](https://www.scopus.com/authid/detail.uri?authorId=7102479102&amp;eid=2-s2.0-0024678788), [Mukherjee KK](https://www.scopus.com/authid/detail.uri?authorId=22963221900&amp;eid=2-s2.0-0024678788) | Seroepidemiological study of Japanese encephalitis in Dimapur, Nagaland | 1989 |
| Am J Trop Med Hyg | [Russell PK](https://www.scopus.com/authid/detail.uri?authorId=35472829000&amp;eid=2-s2.0-0014309298), [Yuill TM](https://www.scopus.com/authid/detail.uri?authorId=7006383747&amp;eid=2-s2.0-0014309298), [Nisalak A](https://www.scopus.com/authid/detail.uri?authorId=7005747529&amp;eid=2-s2.0-0014309298), [Udomsakdi S](https://www.scopus.com/authid/detail.uri?authorId=6602284632&amp;eid=2-s2.0-0014309298), [Gould DJ](https://www.scopus.com/authid/detail.uri?authorId=7201622609&amp;eid=2-s2.0-0014309298), [Winter PE](https://www.scopus.com/authid/detail.uri?authorId=7202002684&amp;eid=2-s2.0-0014309298) | An insular outbreak of dengue hemorrhagic fever. II. Virologic and serologic studies | 1968 |
| J Commun Dis | [Jain SK](https://www.scopus.com/authid/detail.uri?authorId=55461273200&amp;eid=2-s2.0-38649120084), [Kumar K](https://www.scopus.com/authid/detail.uri?authorId=7402675540&amp;eid=2-s2.0-38649120084), [Bhattacharya D](https://www.scopus.com/authid/detail.uri?authorId=7201931914&amp;eid=2-s2.0-38649120084), [Venkatesh S](https://www.scopus.com/authid/detail.uri?authorId=57214394276&amp;eid=2-s2.0-38649120084), [Jain DC](https://www.scopus.com/authid/detail.uri?authorId=7202913108&amp;eid=2-s2.0-38649120084), [Lal S](https://www.scopus.com/authid/detail.uri?authorId=9333798100&amp;eid=2-s2.0-38649120084) | Chikungunya viral disease in district Bhilwara (Rajasthan) India | 2007 |
| Tropical and Geographical Medicine | Adesina AO, Odelola HA | Ecological distribution of Chikungunya haemagglutination inhibition antibodies in human and domestic animals in Nigeria | 1991 |
| J Med Primatol | [Peiris](https://www.semanticscholar.org/author/Joseph-S.-M.-Peiris/32916224) JSM, [Dittus](https://www.semanticscholar.org/author/Wolfgang-P.-J.-Dittus/3735803) WPJ,  [Ratnayake](https://www.semanticscholar.org/author/Chandra-Ratnayake/3921271) C | Seroepidemiology of dengue and other arboviruses in a natural population of toque macaques (Macaca sinica) at Polonnaruwa, Sri Lanka | 1993 |
