# Supplementary material for: Chikungunya seroprevalence in population-based studies: a systematic review and meta-analysis
Source: Arch Public Health. 2023 May 1;81:80. doi: 10.1186/s13690-023-01081-8 (PMC10150504; doi:10.1186/s13690-023-01081-8)
Supplement: Supplementary file 2 — Supplementary Material 2 [file 13690_2023_1081_MOESM2_ESM.docx]

**Additional file 3. Quality analysis of papers.**

| **Author/year** | **sample frame appropriate** | **appropriate way of subjects sampling** | **adequate sample size** | **subjects and the setting described in detail** | **coverage of data analysis** | **valid methods to identify the condition** | **condition measured reliable for all participants** | **appropriate statistical analysis** | **response rate adequate or managed appropriately** | **number of yes** |
| --- | --- | --- | --- | --- | --- | --- | --- | --- | --- | --- |
| Azami 2013 | 1 | 1 | 1 | 1 | 1 | 1 | 1 | 1 | 1 | 9 |
| Cortes-Escamilla 2018 | 1 | 1 | 1 | 1 | 1 | 1 | 1 | 1 | 1 | 9 |
| Cunha 2017 | 1 | 1 | 1 | 1 | 1 | 1 | 1 | 1 | 1 | 9 |
| Dias 2018 | 1 | 1 | 1 | 1 | 1 | 1 | 1 | 1 | 1 | 9 |
| Rodriguez-Barraquer 2015 | 1 | 1 | 1 | 1 | 1 | 1 | 1 | 1 | 1 | 9 |
| Andayi 2014 | 1 | 1 | 0 | 1 | 1 | 1 | 1 | 1 | 1 | 8 |
| Ang 2017 | 1 | 1 | 0 | 1 | 1 | 1 | 1 | 1 | 1 | 8 |
| Barreto 2020 | 1 | 1 | 1 | 1 | 0 | 1 | 1 | 1 | 1 | 8 |
| Humphrey 2019 | 1 | 1 | 1 | 1 | 1 | 1 | 1 | 1 | 0 | 8 |
| Kumar 2011 | 1 | 1 | 1 | 1 | 1 | 1 | 1 | 1 | 0 | 8 |
| LaBeaud 2015 | 1 | 1 | 1 | 1 | 1 | 1 | 1 | 1 | 0 | 8 |
| Laoprasopwattana 2016 | 1 | 1 | 1 | 1 | 1 | 1 | 1 | 1 | 0 | 8 |
| Mease 2011 | 1 | 1 | 1 | 1 | 1 | 1 | 1 | 1 | 0 | 8 |
| Min. Health Nicaragua 2017 | 1 | 1 | 1 | 1 | 1 | 1 | 1 | 1 | 0 | 8 |
| Moro 2010 | 1 | 1 | 1 | 1 | 1 | 1 | 1 | 1 | 0 | 8 |
| Ochieng 2015 | 1 | 1 | 1 | 1 | 1 | 1 | 1 | 1 | 0 | 8 |
| Perisse 2020 | 1 | 1 | 1 | 1 | 1 | 1 | 1 | 1 | 0 | 8 |
| Rogier 2018 | 1 | 1 | 1 | 1 | 1 | 1 | 1 | 1 | 0 | 8 |
| Sissoko 2008 | 1 | 1 | 1 | 1 | 1 | 1 | 1 | 1 | 0 | 8 |
| Sissoko 2008_2 | 1 | 1 | 1 | 1 | 1 | 1 | 1 | 1 | 0 | 8 |
| Solgi 2020 | 1 | 1 | 1 | 1 | 1 | 1 | 1 | 1 | 0 | 8 |
| Somlor 2017 | 1 | 1 | 1 | 1 | 1 | 1 | 1 | 1 | 0 | 8 |
| Vongpunsawad 2017 | 1 | 1 | 1 | 1 | 1 | 1 | 1 | 1 | 0 | 8 |
| Chisenga 2020 | 1 | 1 | 1 | 1 | 0 | 1 | 1 | 1 | 0 | 7 |
| Endale 2020 | 1 | 1 | 0 | 1 | 1 | 1 | 1 | 1 | 0 | 7 |
| Kuan 2016 | 1 | 1 | 1 | 1 | 0 | 1 | 1 | 1 | 0 | 7 |
| Moyen 2014 | 1 | 1 | 0 | 1 | 1 | 1 | 1 | 1 | 0 | 7 |
| Patil 2020 | 1 | 1 | 0 | 1 | 1 | 1 | 1 | 1 | 0 | 7 |
| Queyriaux 2008 | 0 | 1 | 0 | 1 | 1 | 1 | 1 | 1 | 1 | 7 |
| Sharp 2019 | 1 | 1 | 1 | 1 | 0 | 1 | 1 | 1 | 0 | 7 |
| Anjos 2020 | 1 | 1 | 0 | 1 | 0 | 1 | 1 | 1 | 0 | 6 |
| Hennesey 2018 | 0 | 1 | 0 | 1 | 1 | 1 | 1 | 1 | 0 | 6 |
| Kuniholm 2006 | 0 | 1 | 0 | 1 | 1 | 1 | 1 | 1 | 0 | 6 |
| Schwarz 2012 | 0 | 1 | 0 | 1 | 1 | 1 | 1 | 1 | 0 | 6 |
| Sergon 2007 | 1 | 1 | 0 | 1 | 0 | 1 | 1 | 1 | 0 | 6 |
| Sergon 2008 | 1 | 1 | 0 | 1 | 0 | 1 | 1 | 1 | 0 | 6 |
| Sow 2020 | 1 | 1 | 0 | 1 | 0 | 1 | 1 | 1 | 0 | 6 |
| Ster 2020 | 1 | 1 | 0 | 1 | 0 | 1 | 1 | 1 | 0 | 6 |
| Vilibic-Cavlek 2015 | 1 | 1 | 0 | 0 | 1 | 1 | 1 | 1 | 0 | 6 |
| Gerardin 2018 | 1 | 1 | 0 | 0 | 0 | 1 | 1 | 1 | 0 | 5 |
| Kama 2019 | 0 | 0 | 0 | 1 | 1 | 1 | 1 | 1 | 0 | 5 |
| Kokernot 1960 | 0 | 0 | 0 | 1 | 1 | 1 | 1 | 1 | 0 | 5 |
| Martins Netto 2017 | 0 | 0 | 0 | 1 | 1 | 1 | 1 | 1 | 0 | 5 |
| Nabli 1970 | 0 | 0 | 0 | 1 | 1 | 1 | 1 | 1 | 0 | 5 |
| Porter 2004 | 0 | 1 | 0 | 1 | 0 | 1 | 1 | 1 | 0 | 5 |
| Dellagi 2016 | 0 | 0 | 0 | 1 | 0 | 1 | 1 | 1 | 0 | 4 |
| Demanou 2010 | 0 | 0 | 0 | 1 | 0 | 1 | 1 | 1 | 0 | 4 |
| Farnon 2010 | 0 | 0 | 0 | 1 | 0 | 1 | 1 | 1 | 0 | 4 |
| Gallian 2017 | 0 | 0 | 0 | 1 | 0 | 1 | 1 | 1 | 0 | 4 |
| Gay 2016 | 0 | 0 | 0 | 1 | 0 | 1 | 1 | 1 | 0 | 4 |
| Kanomitsu 1979 | 0 | 0 | 0 | 1 | 0 | 1 | 1 | 1 | 0 | 4 |
| Padbidri 2002 | 0 | 0 | 0 | 1 | 0 | 1 | 1 | 1 | 0 | 4 |
| Sutherland 2011 | 0 | 0 | 0 | 1 | 0 | 1 | 1 | 1 | 0 | 4 |
| Aubry 2015 | 0 | 0 | 0 | 0 | 0 | 1 | 1 | 1 | 0 | 3 |
| Aubry 2018 | 0 | 0 | 0 | 0 | 0 | 1 | 1 | 1 | 0 | 3 |
| Aubry 2020 | 0 | 0 | 0 | 0 | 0 | 1 | 1 | 1 | 0 | 3 |
| Bacci 2015 | 0 | 0 | 0 | 0 | 0 | 1 | 1 | 1 | 0 | 3 |
| Barakat 2016 | 0 | 0 | 0 | 0 | 0 | 1 | 1 | 1 | 0 | 3 |
| Bowen 1973 | 0 | 0 | 0 | 0 | 0 | 1 | 1 | 1 | 0 | 3 |
| Eligio-Garcia 2020 | 0 | 0 | 0 | 0 | 0 | 1 | 1 | 1 | 0 | 3 |
| Gabor 2016 | 0 | 0 | 0 | 0 | 0 | 1 | 1 | 1 | 0 | 3 |
| Inziani 2020 | 0 | 0 | 0 | 1 | 0 | 1 | 0 | 1 | 0 | 3 |
| Ivanov 1992 | 0 | 0 | 0 | 0 | 0 | 1 | 1 | 1 | 0 | 3 |
| Atalay 2017 | 0 | 0 | 0 | 0 | 0 | 1 | 1 | 0 | 0 | 2 |
