# Supplementary figures and images for: Chikungunya seroprevalence in population-based studies: a systematic review and meta-analysis
Source: Arch Public Health. 2023 May 1;81:80. doi: 10.1186/s13690-023-01081-8 (PMC10150504; doi:10.1186/s13690-023-01081-8)

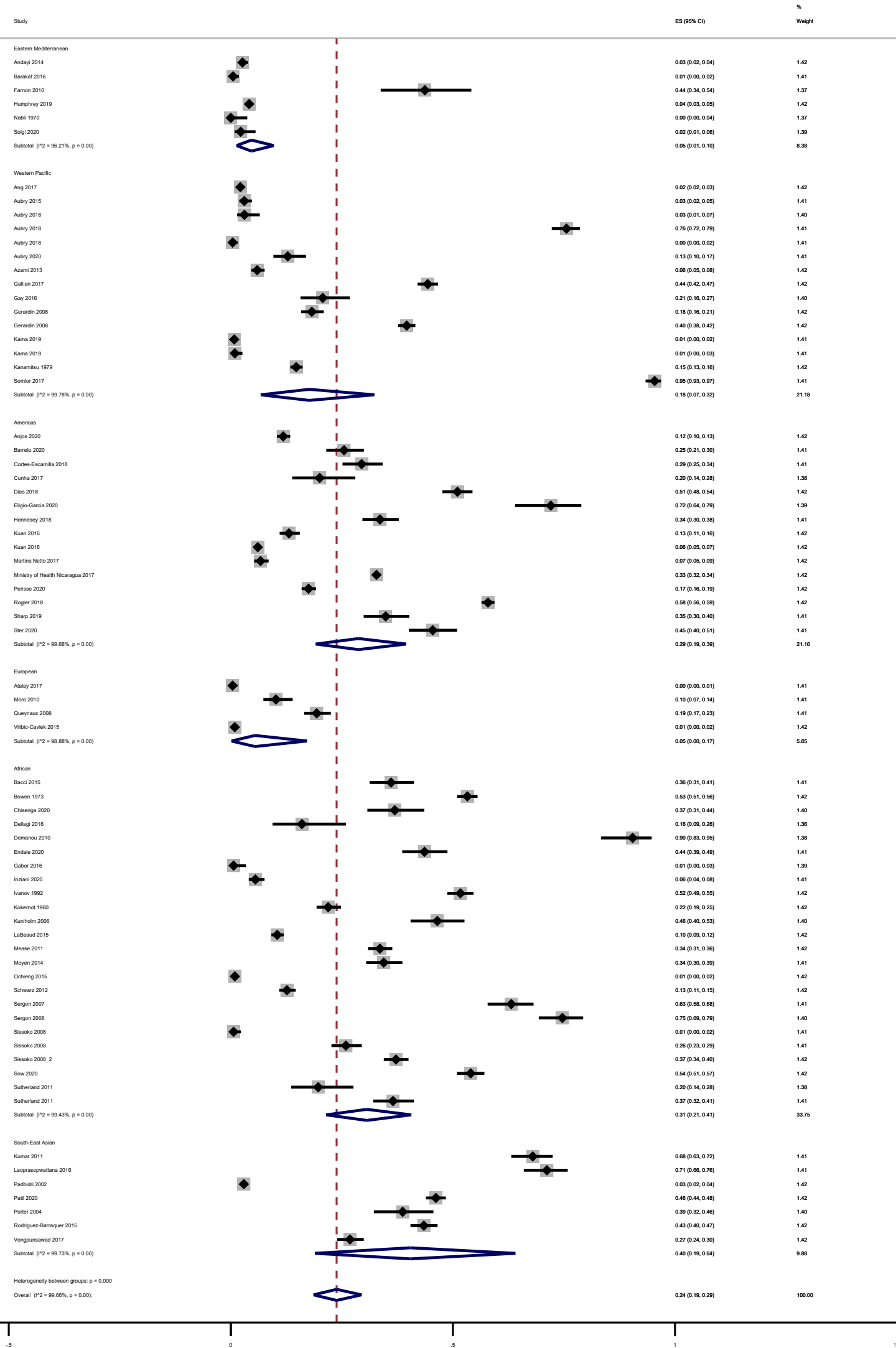

Supplement: Supplementary file 4 — Supplementary Material 4 [file 13690_2023_1081_MOESM4_ESM.pdf]
